# Decoding information in the human hippocampus: A user's guide

**DOI:** 10.1016/j.neuropsychologia.2012.07.007

**Published:** 2012-11

**Authors:** Martin J. Chadwick, Heidi M. Bonnici, Eleanor A. Maguire

**Affiliations:** Wellcome Trust Centre for Neuroimaging, Institute of Neurology, University College London, 12 Queen Square, London WC1N 3BG, UK

**Keywords:** fMRI, Hippocampus, Decoding, MVPA, Autobiographical memory, Episodic memory, Navigation, Scenes

## Abstract

Multi-voxel pattern analysis (MVPA), or ‘decoding’, of fMRI activity has gained popularity in the neuroimaging community in recent years. MVPA differs from standard fMRI analyses by focusing on whether information relating to specific stimuli is encoded in patterns of activity across multiple voxels. If a stimulus can be predicted, or decoded, solely from the pattern of fMRI activity, it must mean there is information about that stimulus represented in the brain region where the pattern across voxels was identified. This ability to examine the representation of information relating to specific stimuli (e.g., memories) in particular brain areas makes MVPA an especially suitable method for investigating memory representations in brain structures such as the hippocampus. This approach could open up new opportunities to examine hippocampal representations in terms of their content, and how they might change over time, with aging, and pathology. Here we consider published MVPA studies that specifically focused on the hippocampus, and use them to illustrate the kinds of novel questions that can be addressed using MVPA. We then discuss some of the conceptual and methodological challenges that can arise when implementing MVPA in this context. Overall, we hope to highlight the potential utility of MVPA, when appropriately deployed, and provide some initial guidance to those considering MVPA as a means to investigate the hippocampus.

## Highlights

► Multi-voxel pattern analysis (MVPA) is increasingly used to analyse fMRI data. ► We review the relatively few studies that have used MVPA to examine the hippocampus. ► We consider methodological challenges related to implementing this approach. ► We conclude that fMRI MVPA can offer new insights into the role of the hippocampus.

## 1 Introduction

It has been clear for many decades that the hippocampus is critical for memory. Lesions to this structure leave afflicted patients with dense anterograde amnesia and significant retrograde memory loss for their personal experiences (Scoville & Milner, 1957; Mayes, 1988; Spiers, Maguire, & Burgess, 2001; Mayes & Montaldi, 2001; Mayes & Roberts, 2001; Mayes, 2008; Winocur & Moscovitch, 2011). While it is now widely accepted that these episodic memories are supported by a distributed set of brain regions (Maguire, 2001; Svoboda, McKinnon, & Levine, 2006), nevertheless, the hippocampal contribution is still regarded as key.

Computational models posit the existence of some form of memory representation within the hippocampus that is vital for retrieving an entire memory, the constituent elements of which may be distributed in cortical areas (Marr, 1971; Treves & Rolls, 1994; O'Reilly & McClelland, 1994; McClelland & Goddard, 1996; O'Reilly & Rudy, 2000). The theoretical nature of this hippocampal memory trace has been modelled over the years, and related computations such as pattern separation and pattern completion have been extensively studied in the rodent hippocampus (Vazdarjanova & Guzowski, 2004; Lee, Yoganarasimha, Rao, & Knierim, 2004; Leutgeb, Leutgeb, Treves, Moser, & Moser, 2004; Wills, Lever, Cacucci, Burgess, & O'Keefe, 2005; Leutgeb, Leutgeb, Moser, & Moser, 2007). Overall, this body of work has produced detailed predictions regarding the expected nature of memory representations within the hippocampus. To date, however, it has proved difficult to conduct compelling tests of these predictions in the human hippocampus, and so it remains unclear precisely how individual episodic memories are represented by neuronal populations in the human hippocampus.

This dearth of knowledge concerning human hippocampal memory representations is in part a result of the dominant methodological approaches in human cognitive neuroscience. Neuropsychological studies have been invaluable for mapping out the specific patterns of mnemonic sparing and deficits that arise following damage to the hippocampus and other structures (Scoville & Milner, 1957; Mayes, 1988; Spiers et al., 2001; Mayes & Montaldi, 2001; Mayes & Roberts, 2001; Maguire, Nannery, & Spiers, 2006; Mayes, 2008; Winocur & Moscovitch, 2011), but cannot inform directly about the neuronal representation of specific memory traces. Functional neuroimaging, and particularly functional MRI (fMRI), has also proved effective for localising a wide range of cognitive functions to specific brain regions whilst also highlighting the importance of network activity (e.g., Bullmore & Sporns, 2009; Yarkoni, Poldrack, Nichols, Van Essen, & Wager, 2011). The standard method of fMRI analysis, and one that has dominated this field, is the mass-univariate approach. Mass-univariate analysis involves creating a model of the experimental design that is fitted to the fMRI BOLD response at each voxel independently (a voxel is the smallest unit we can measure in a 3D brain image volume), the aim being to find activity in voxels that consistently shows a relationship with the experimental design. This activity is spatially smoothed for every subject, and the activity from each individual is normalized to the same template in order to discover regions that show global changes in BOLD in response to the experimental variable at the group level (Frackowiak et al., 2004). This method is highly effective for investigating many types of brain function, but is generally insensitive to fine-grained levels of representation such as individual memory traces.

To illustrate this point further, consider a paradigm where a participant is recalling two individual episodic memories and two individual semantic memories (we will assume that one trial of each memory provides sufficient power for this hypothesised analysis, although this in not actually the case in reality). Using a mass-univariate analysis, we first compare the hippocampal BOLD response evoked by the two episodic memories to that evoked by the semantic memories and, as expected, discover that episodic retrieval produces a greater fMRI response (Maguire, 2001; Svoboda et al., 2006). Next we want to look for activation that is specific to each episodic memory, so we directly contrast one episodic memory with the other. However, when we compare them, we find no difference, as each memory has evoked a similar increase in BOLD response across the hippocampus. Consequently, while this method has proved invaluable for establishing the involvement of various regions in memory encoding and retrieval, including the hippocampus, it does not permit the investigation of individual memory representations.

In recent years, an alternative approach to fMRI analysis has emerged which exploits the intrinsically multivariate nature of fMRI data. The motivation for this change stems from the belief that there may be information present in the distributed pattern of activation across voxels that is missed when looking at each voxel independently as in the mass-univariate method (Haynes & Rees, 2006; Norman, Polyn, Detre, & Haxby, 2006). This type of multivariate method is commonly known as multi-voxel pattern analysis (MVPA), or decoding. A clear demonstration of the potential of MVPA was provided by Haxby et al. (2001), who found that neural representations of object categories, such as places and faces, were more widely distributed and overlapping within the ventral temporal cortex than had been thought previously. Importantly, they examined specific regions where the individual voxels (using a mass-univariate approach) responded strongly to one category or another, and found that within these supposedly category-selective regions, there still existed considerable information in the distributed pattern of activation about the non-preferred category. This illustrates the complementary nature of the information offered by mass-univariate and MVPA analyses, and suggests that MVPA may be more sensitive to the presence of information about specific representations such as object categories. Since this early study, MVPA has been applied in a wide range of cognitive domains including perception (Haynes & Rees, 2005; Kamitani & Tong, 2005), emotion (Peelen, Atkinson, & Vuilleumier, 2010; Baucom, Wedell, Wang, Blitzer, & Shinkareva, 2011), and decision-making (Kahnt, Heinzle, Park, & Haynes, 2011).

**Fig. 1:**
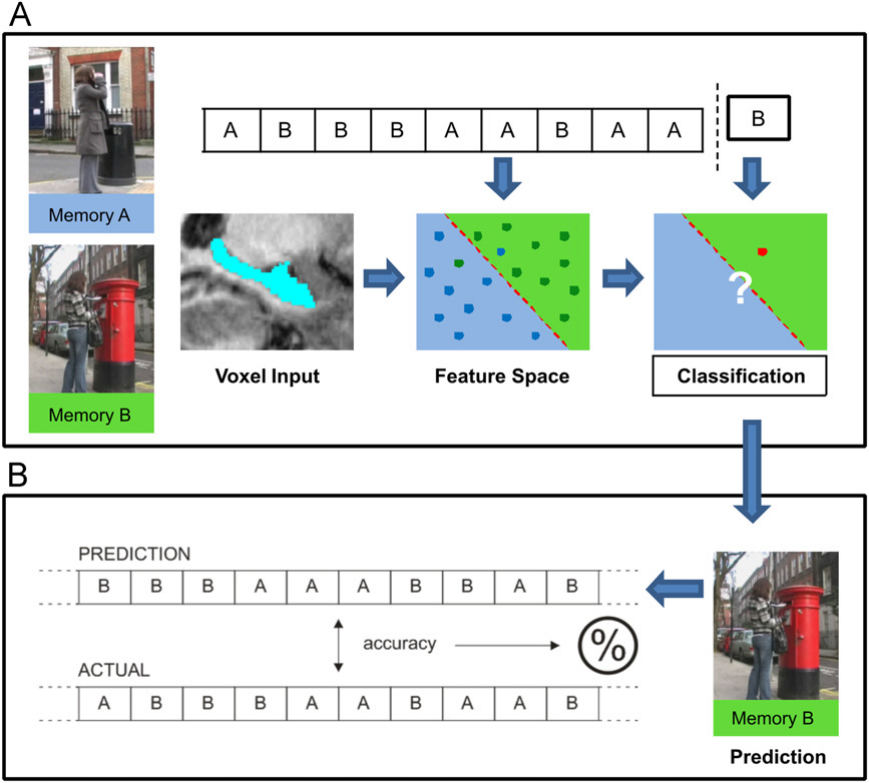
An illustration of MVPA classification. (A) In this example, the analysis involves trying to classify two episodic-like memories from patterns of voxel activity in the hippocampus. Each memory is recalled five times, and the activity related to each recall trial is extracted. The string of letters represents activity from the hippocampus on each trial, labelled as either Memory A or B. The full dataset is split into a “training” set and a “testing” set, in this case assigning a single trial to the test dataset. Using the training set, an MVPA classifier is trained to differentiate memories A and B based on the patterns of activation in the hippocampus, and then tested on the test set. (B) In this example the test trial was classified as Memory B, which was a correct prediction. In a leave-one-out cross-validation, this process would be repeated ten times, each time leaving out a different trial as the test dataset. This cross-validation therefore yields a predicted label for every data trial in the analysis, which can then be compared against the real labels to produce an overall classification accuracy.

A simplified example of a standard MVPA analysis is shown in Fig. 1, which is loosely based on the study by Chadwick, Hassabis, Weiskopf, and Maguire (2010). In this instance, the participant has vividly recalled two episodic memories (Memories A and B) five times each during scanning. The aim of the analysis is to try and find unique patterns of voxel activation that consistently map on to each of the two memories across the different recall trials. In order to do this, the standard MVPA approach involves setting aside a portion of the data (in this case a single trial) to be the “testing” dataset. The remaining nine trials are used to train an MVPA algorithm (such as a support vector machine (SVM) classifier or linear discriminant function—there are many choices of algorithm, which we discuss in more detail in Section 3). This training process involves finding an optimal “decision boundary” within the high-dimensional space of the features (the individual recall trials), which best separates the Memory A trials (in green) from the Memory B trials (in blue). The trial which was kept aside from the training set can now be given to the trained MVPA algorithm, which produces a predicted class label based on which side of the decision boundary the trial falls on. In this example the test trial was classified as Memory B, which was a correct prediction. In order to assess the information contained within the entire dataset, the standard approach used is cross-validation, whereby the procedure described above is repeated many times, each time leaving out a different portion of the data as the test set. In this example, we would repeat the process ten times in total, each time leaving out one of the ten trials in the test set. Overall this produces a set of ten predicted class labels, one for each of the data trials. This predicted set can then be compared to the actual class labels of the data, producing a percent correct accuracy score for the entire dataset. This is then compared to chance level performance, which in this example is 50% as there are two memories.

The example above involves a categorical MVPA problem, where the algorithm is required to produce a categorical decision (is it Memory A or B?). This is currently the most common type of MVPA used in fMRI analysis, and is generally referred to as MVPA classification. This is the form of MVPA used in all of the experiments we describe in Section 2. However, it is also possible to use MVPA for the investigation of continuous variables, although generally this requires the use of different algorithms (such as support vector regression).

One important point to clarify is precisely what type of analysis constitutes MVPA as opposed to more general multivariate analyses, such as independent or principal component analysis (ICA/PCA). As already mentioned, there are many types of MVPA analysis available, and what links these types of analysis is not the specific classification procedure outlined above, as some analyses such as representational similarity analysis (Kriegeskorte, Mur, & Bandettini, 2008a; Kriegeskorte et al., 2008b) or multivariate Bayesian decoding (Friston et al., 2008) do not require this protocol. Rather, the critical defining factor is that all MVPA methods aim to find an explicit mapping between an experimental variable and the multivariate data (thus, like all other methods of fMRI, it is based on correlational evidence). This stands in contrast to multivariate methods like ICA or PCA in which there is no explicit mapping. Interestingly, this means that in theory one could consider a simple multiple regression analysis to be a form of MVPA, if it were used to map multivariate voxel responses to an experimental variable. However, in practice this is rarely possible, as multiple regression is only viable when the number of explanatory variables (in this case voxels) is less than the number of data points. This is highly limiting when dealing with fMRI data, where we are likely to want to mine information from (at the very least) hundreds of voxels. Hence the reason for the extensive use of more complex MVPA algorithms such as the SVM, which deal very well with cases where there are many more data features than stimulus trials.

Overall it is clear that MVPA provides a potentially powerful method for investigating neural information at the level of individual memory representations, and thus far a few studies have used this approach to investigate cortical memory representations (see Rissman and Wagner (2012) for a comprehensive review). Despite the important insights gained from these studies of the neocortex, to date, there have been few studies using MVPA to investigate memory traces specifically within the hippocampus. Given the extensive theoretical frameworks that exist for the hippocampus, we argue that the hippocampus provides an excellent target for hypothesis-driven MVPA studies. Indeed, such an approach may prove to be vital for forming an empirical bridge between the detailed computational accounts of memory (Marr, 1971; Treves & Rolls, 1994; O'Reilly & McClelland, 1994; McClelland & Goddard, 1996; O'Reilly & Rudy, 2000) and the biological substrates of more complex forms of human memory.

The purpose of this review is two-fold. First, we will review the small number of published studies that have so far used MVPA to investigate memory representations within the human hippocampus. The aim of this section (Section 2) is to demonstrate that MVPA can be used to investigate important, hypothesis-driven questions that are not amenable to standard mass-univariate fMRI analysis. Following this, in Section 3 we discuss some of the conceptual and methodological challenges that can arise when implementing MVPA in this context by addressing frequently-asked-questions that we typically experience in relation to our MVPA research. This section is primarily aimed at hippocampal researchers who wish to try MVPA analysis, although the issues discussed in this section are in fact applicable to any study utilising MVPA with fMRI. Overall, our goal is to promote greater application of MVPA to the study of the hippocampus, as we believe that appropriate deployment of this method has the potential to provide important new perspectives on the function of the human hippocampus.

## 2 Using MVPA to investigate the hippocampus

Here for convenience, but primarily because there are just so few published MVPA studies that have investigated the hippocampus specifically, we now consider four of our own experiments to illustrate that novel insights can arise from application of MVPA in this context. In all cases we acquired high-resolution fMRI data (Carr, Rissman, & Wagner, 2010) using a 3T MRI scanner. High-resolution data result in smaller and more numerous voxels which, if the interest is in examining patterns of activity across voxels, allows one to discern finer patterns more readily. The resolution of our images was 1.5 mm (isotropic voxels) which we acquired by focusing on the medial temporal lobes (MTL).

### 2.1 Decoding spatial information in the human hippocampus

**Fig. 2:**
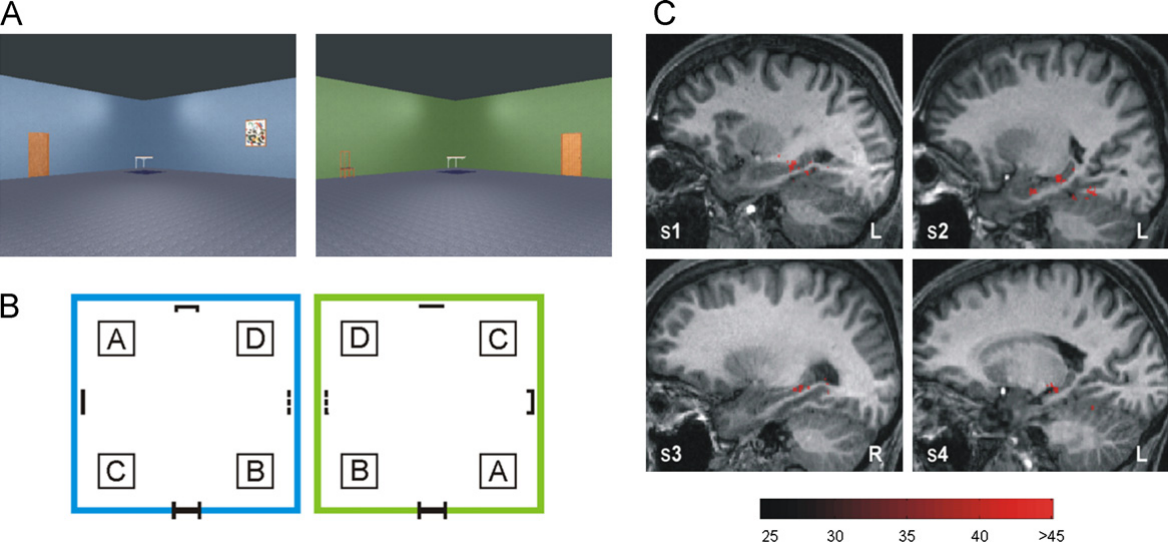
Decoding spatial locations in the hippocampus (Hassabis et al., 2009). (A) Two separate and distinct virtual environments were used, a blue room and a green room. Each room was 15 m^2^ and contained four “target” positions, which participants were instructed to navigate between as quickly and accurately as possible following extensive pre-scan training. (B) Schematic of the room layouts with the four target positions, labelled A, B, C, and D (these were not labelled in the experiment). These targets were visually delineated by identical cloth rugs placed on the floor at those positions. Single objects (door, chair, picture, and clock with different exemplars per room but of similar size and colour) were placed along the centre of each wall to act as orientation cues. Identical small tables were placed in each of the four corners in order to help visually delineate the wall boundaries. (C) Results for each of the four participants, showing the classification accuracies of the voxels at the centre of searchlights that discriminated between all four target positions in the same room significantly better than chance (25%). The red bar indicates percentage accuracy values. These results clearly demonstrate that voxels in the body-posterior hippocampus contained a significant degree of spatial location information for each participant. (For interpretation of the references to color in this figure legend, the reader is referred to the web version of this article.)

The first use of MVPA to explore representations specifically in the human hippocampus was in a study by Hassabis et al. (2009). The hippocampus has long been known to play a crucial role in the representation of space, and particularly allocentric spatial location as exemplified by the existence of “place cells” in both rodents (O'Keefe & Dostrovsky, 1971; O'Keefe & Nadel, 1978; see also Burgess, Maguire, & O'Keefe, 2002) and humans (Ekstrom et al., 2003). The central aim of the Hassabis et al. (2009) study was to determine the feasibility of using MVPA to decode the location of participants in a virtual environment. Participants controlled their movement within a virtual room (Fig. 2A) while undergoing scanning, and were required to navigate between all four corners of the room in a pseudo-random order. This was repeated across multiple blocks within two separate virtual rooms (Fig. 2B). Importantly, whenever the participants reached a target location, there was a period where their view within the virtual room automatically tilted down to look at a patch of carpet which was visually matched across the four target locations; there was then a visual countdown to the start of the next trial. The activity from these periods was extracted and used in the MVPA analysis, meaning that the direct visual input was exactly matched for each of the four locations within each room. Nevertheless, using an MVPA analysis constrained to the MTL, it was possible to decode these four locations from patterns of fMRI activity across voxels in the hippocampus in each of four participants (Fig. 2C).

In a second analysis, Hassabis et al. (2009) found that information about the two different virtual environments (regardless of specific spatial location) was present within the posterior parahippocampal cortex, but not the hippocampus, suggesting that these two regions may play dissociable roles in the representation of spatial information. A subsequent study by Rodriguez (2011) applied an MVPA classifier to data collected from participants navigating around a circular virtual environment, and also found evidence for the existence of specific spatial representations within the hippocampus. Put together, these results demonstrate that highly abstracted representations of space are present and detectable from patterns of fMRI activity in the human hippocampus. More generally, these studies demonstrated that the hippocampus is a viable target for MVPA studies.

It is important to note that Hassabis et al. (2009) conducted an equivalent mass-univariate analysis of these data, and found no significant results even at liberal statistical thresholds. As outlined in Section 1, this failure to find a result is not surprising, as we would expect the hippocampus to show a similar global change in activation across the four individual locations, and so be unable to differentiate between them. The same point applies to each study described in this section, where a mass-univariate analysis failed to produce significant results, in contrast to MVPA of the same data. This difference demonstrates the potential power of the MVPA method for certain types of question.

### 2.2 Decoding episodic memories

In addition to its role representing space and in navigation, the hippocampus is also crucial for episodic/autobiographical memory (Scoville & Milner, 1957; Mayes, 1988; Spiers et al., 2001; Mayes & Montaldi, 2001; Mayes & Roberts, 2001; Mayes, 2008; Winocur & Moscovitch, 2011). FMRI studies using mass-univariate analyses have demonstrated robust activation of the hippocampus during autobiographical memory recall (see Maguire (2001), Svoboda et al. (2006) for reviews), but do not inform about the representation of each individual memory. Due to this constraint, we know little about the underlying neural code of episodic memories within the hippocampus, although numerous theories abound (Marr, 1971; O'Reilly & McClelland, 1994; McClelland & Goddard, 1996; O'Reilly & Rudy, 2000). A critical question, therefore, is whether it is possible to use MVPA to differentiate individual episodic memories from patterns of activity. If so, this could open up new opportunities for exploring the neural code underlying human episodic memory.

**Fig. 3:**
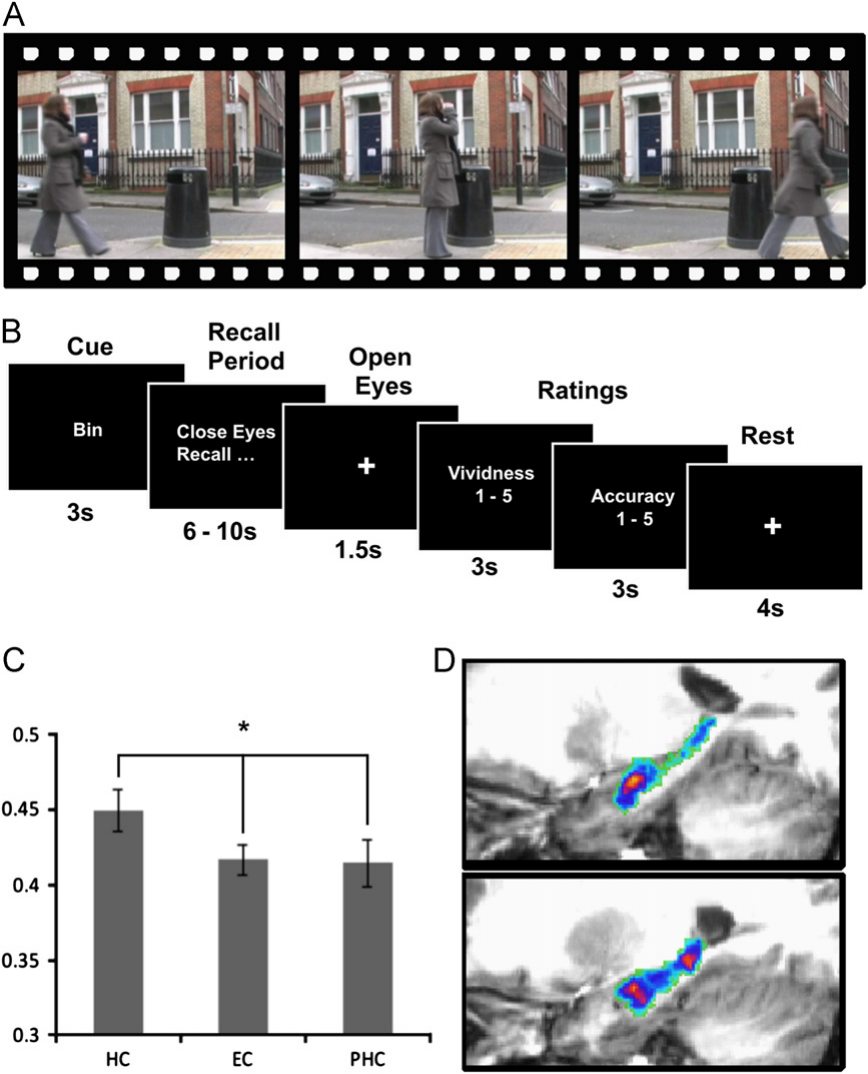
Decoding episodic-like memories in the hippocampus (Chadwick et al., 2010). (A) Still photographs taken from one of the film clips viewed during pre-scan training. The clip depicted a woman taking a drink from a disposable coffee cup and then putting it in a bin (trashcan). (B) Timeline of an example trial during fMRI scanning. (C) Group mean MVPA classification accuracy as a proportion, with standard error bars for the hippocampus (HC), entorhinal cortex (EC), and posterior parahippocampal cortex (PHC). Classification accuracy for all three areas was significantly above chance level performance (0.33), with HC accuracy significantly greater than both EC and PHC (*p<0.05). (D) Frequency heatmap showing the overlap in the location of episodic information in the hippocampus across participants. Regions in bilateral anterior hippocampus and posterior right hippocampus had significantly more overlap than would be expected by chance.

Chadwick et al. (2010) explored this question using a simple paradigm where ten participants were shown three short video clips of everyday events in a pre-scan session (Fig. 3A). They then practised vividly recalling each of these clips until they could do so vividly and consistently on each recall. Once fully trained, the participants repeatedly recalled each of the three clips as vividly as possible during fMRI. On each trial of scanning, one of the three memories was cued verbally, and the participant was then instructed to close their eyes and recall that memory (see Fig. 3B for timeline of an example trial). Following this recall period, the participant was asked to provide a rating of the vividness and accuracy of that recall trial on a five point scale, and any trials with ratings lower than 3/5 were discarded from the analysis, ensuring that all trials entered into the MVPA analysis were vivid memories. In addition to this cued condition, a free recall condition was also included, in which the participants themselves decided which memory to recall on each trial. Notably, the results were highly consistent across both cued and free recall conditions.

Three regions of interest (ROIs) were defined within the MTL—the hippocampus, entorhinal cortex, and posterior parahippocampal cortex. The data related to each recall trial were extracted from each of these ROIs, and assigned a label based on the particular memory being recalled on that trial (e.g., Memories A–C). Chadwick et al. (2010) then applied an MVPA algorithm incorporating a searchlight feature selection step to this labelled data. The purpose of feature selection is to try and reduce the noise within the dataset, and boost the power of the MVPA analysis (see Section 3.4 for more details). Overall this analysis produced a single accuracy value for each ROI, which was then compared against the accuracy expected by chance (33% in this case, as there were three memories). In the first instance they found that all three MTL regions produced classification results that were significantly above chance, showing that it is possible to detect information about individual episodic-like memories from activity patterns within the hippocampus and surrounding MTL regions (Fig. 3C). Importantly, they also found that classification accuracy for the hippocampus was significantly better than for the other MTL regions, suggesting that episodic-like memories may be more distinct within the hippocampus than the surrounding cortex, in line with evidence from non-humans and computational modelling (O'Reilly & McClelland, 1994; Treves & Rolls, 1994; McClelland & Goddard, 1996; O'Reilly & Rudy, 2000).

In addition to looking at the overall classification accuracy within each ROI, it was also possible to investigate whether there was intra-hippocampal bias in terms of where information about specific memories was located across our group of participants. To do this Chadwick et al. (2010) took the voxels considered to be most informative from the feature selection step of the classification, and used these to form an “information map” for each participant. Visual inspection of the data suggested a degree of consistency in the location of information across individuals, particularly in the anterior hippocampus. To investigate this they normalized the individual information maps and added them together to create a frequency heatmap (Fig. 3D), where the colour of each voxel represented the number of participants' information maps that overlapped on that voxel. They found significantly more overlap than would be expected by chance in three regions—bilateral anterior and right posterior hippocampus. This result suggests that episodic information is not randomly distributed across the hippocampus, but instead is focused within certain regions. Overall, the results of this study demonstrate that MVPA can be applied to hippocampal representations of episodic-like memories.

### 2.3 Overlapping episodic memories and pattern separation

The previous study involved episodic-like memories but did not allow any conclusions to be drawn regarding the specific representational content of the underlying memories. This is because each memory differed along a variety of dimensions, including the identity of the people involved, the actions performed and the spatial contexts. Consequently it was not possible to determine exactly how the episodes were represented within the hippocampus, or precisely what aspect of the episodes was being detected by the MVPA classification technique. In a further study, MVPA was used to probe the representational properties of episodic-like memories within the hippocampus, and in particular when those memories contained a high degree of overlap in terms of their constituent elements (Chadwick, Hassabis, & Maguire, 2011).

Given that our daily lives usually involve encounters with a relatively limited range of experiences, the episodic memories that we form often contain a large degree of overlap. Nevertheless, most of the time we are able to remember each event as a distinct episode. The hippocampus has long been posited as the critical brain structure involved in separating overlapping episodes into unique representations, which then allows them to be stored as distinct episodic memory traces (Marr, 1971; Treves & Rolls, 1994; O'Reilly & McClelland, 1994; McClelland & Goddard, 1996; O'Reilly & Rudy, 2000). This idea has a strong grounding in the anatomy of the hippocampus and in the rodent literature (Vazdarjanova & Guzowski, 2004; Lee et al., 2004; Leutgeb et al., 2004; Wills et al., 2005; Leutgeb et al., 2007), but empirical evidence for the involvement of pattern separation in complex episodic memory in the human hippocampus remains scarce.

**Fig. 4:**
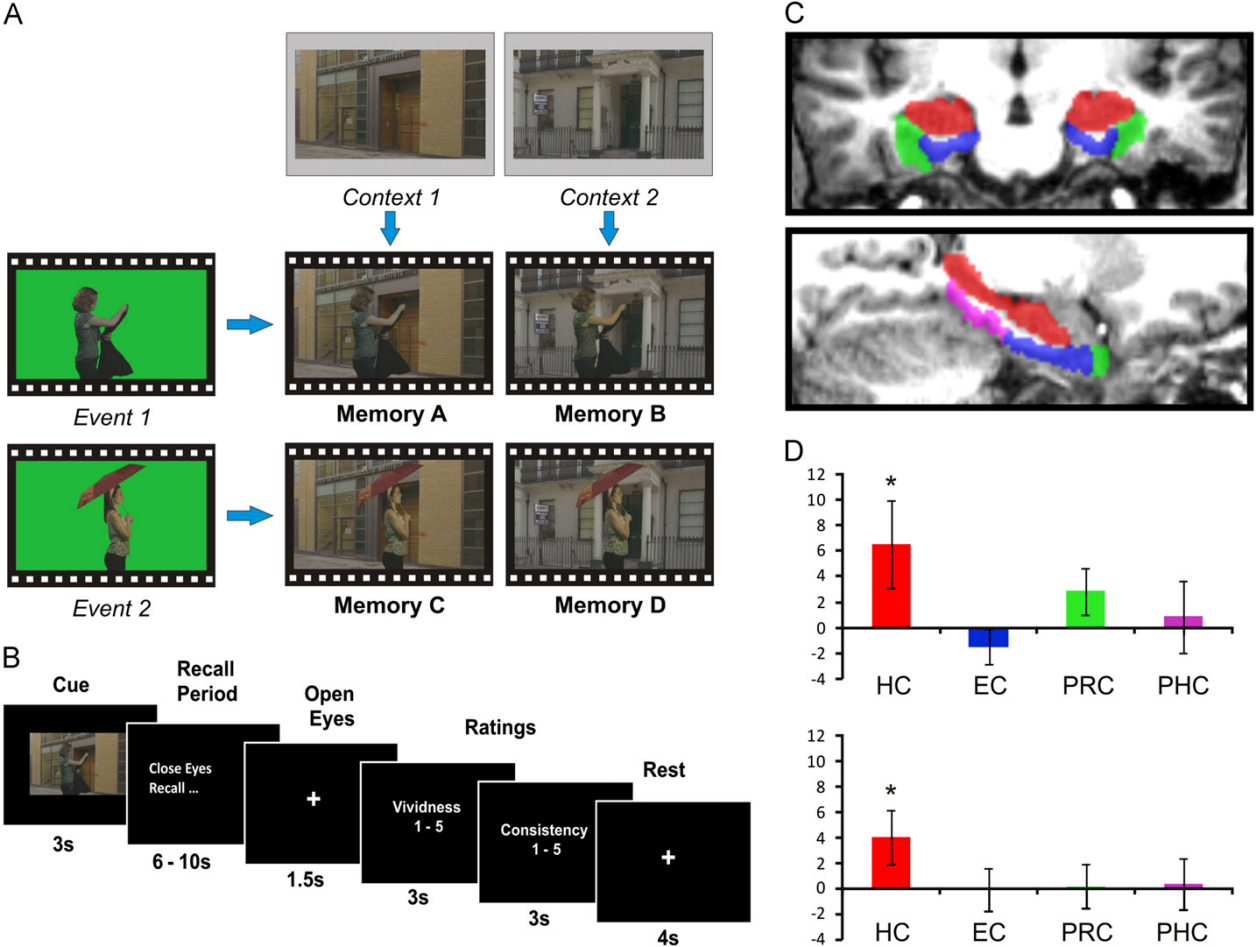
Decoding overlapping episodic-like memories (Chadwick et al., 2011). (A) The stimuli: two events were filmed against a green-screen background (left panels). Each clip was 7 s long and involved a short series of actions performed by a single female actress. In the first, a woman walked into shot, removed her jacket and placed it over her arm. In the second, a woman walked into shot, took out and put up an umbrella. The two events were superimposed on two different spatial contexts (see contexts in uppermost panels) in order to create four movies which included all four combinations of event content and spatial context (see panels Memories A–D). These stimuli ensured that the memories would be dynamic and episodic-like in nature, whilst being fully controlled in terms of the event content and spatial context of each memory. (B) Timeline of an example trial during fMRI scanning. On each trial, one of the four episodes was cued with a still photograph taken from the movie. Following this cue, participants were instructed to close their eyes and recall the episode as vividly and accurately as possible. (C) Segmented regions of interest in the medial temporal lobe of one of the participants shown in the coronal plane (upper panel) and sagittally (lower panel). The hippocampus (HC) is shown in red, entorhinal cortex (EC) in blue, perirhinal cortex (PRC) in green, and the posterior parahippocampal cortex (PHC) in magenta. (D) Group mean MVPA classification results for each of the four MTL regions are displayed for the four-way classification analysis (upper graph), and the spatial context classification analysis (lower graph). Results are displayed as percentage accuracy above chance (*p<0.05), with standard error bars. In both analyses, only the HC results are significantly above chance. (For interpretation of the references to color in this figure legend, the reader is referred to the web version of this article.)

Chadwick et al. (2011) applied MVPA methods to the study of highly overlapping episodic-like memories in order to determine whether it is possible to detect unique memory traces from patterns of activity within the human hippocampus. The overlapping information in the episodes was a critical aspect of this study, as it was important to ensure that no episode could be uniquely specified by any single constituent element within it. In order to create such fully controlled stimuli, two brief action events were filmed against a green-screen background. Each of these events was then superimposed onto two spatial contexts, creating four episodes which included every combination of both events and both contexts (Fig. 4A). As the four episodes completely overlapped with one another in terms of their constituent elements, any successful differentiation of the four memories from patterns of activation must be due to the presence of some unique, conjunctive representation of each episode.

In a pre-scan session, fifteen participants viewed the four movies, and then practiced vividly recalling all four of them until they could do so vividly and accurately each time. Following training, they vividly recalled each episode numerous times during fMRI scanning (Fig. 4B). The critical analysis involved determining whether it was possible to differentiate the four overlapping memories based on patterns of activity within the hippocampus, which would therefore provide evidence that the hippocampus supports unique representations of highly overlapping episodes. Four ROIs within the MTL were delineated, the hippocampus, entorhinal cortex, perirhinal cortex, and posterior parahippocampal cortex (Fig. 4C), and a four-way MVPA classification analysis was applied to each. Only the classifier operating on hippocampal voxels showed significant levels of classification when compared to chance (Fig. 4D), demonstrating that even when memories are completely overlapping with one another in terms of their constituent elements, the hippocampus still contains distinct representations. This result is consistent with the idea that the hippocampus plays a central role in pattern separating overlapping episodes into distinct representations. Notably, when the analysis was repeated after controlling for the different number of voxels in each ROI, the same results pertained, demonstrating that this is not merely an artefact of MTL regions differing in size.

An important aspect of this particular paradigm was that it allowed further inferences to be made about the representations in the hippocampus, in particular, about the representation of spatial context. Although it is well established that the hippocampus plays a central role in spatial representation O'Keefe & Dostrovsky, 1971; O'Keefe & Nadel, 1978; Burgess et al., 2002; Hassabis, Kumaran, Vann, & Maguire, 2007; Hassabis et al., 2009), little is known about the representation of spatial information that forms part of a discrete episodic memory, particularly when that spatial information might be shared across different memories. In order to investigate this issue, Chadwick et al. (2011) performed a further analysis to determine whether there was any evidence that the hippocampus contains general representations of the spatial context of events during episodic recall. As each spatial context was shared by two different memories in this study, this question could be addressed by training an MVPA classifier to differentiate two memories which had the same event content, but different spatial context, and then testing this classifier on the remaining two memories. The only information in common between these two sets of memories was the background spatial context, so any successful classification must be due to some kind of generalised spatial representation. This analysis was applied to all four ROIs, and again, the only significant classification result was for the hippocampus (Fig. 4D). This finding is consistent with evidence suggesting that the hippocampus is critical for spatial representation, but is the first to demonstrate that even during the recall of episodic memories, the hippocampus maintains a distinct representation of relevant spatial environments that might be shared across different episodes. Put together, this set of findings suggests that the hippocampus is capable of supporting at least two different types of representation—each memory has a unique representation created through a process of pattern separation, and at the same time, spatial backdrops that are common to different memories are also represented in the hippocampus.

### 2.4 Decoding overlapping scene representations

**Fig. 5:**
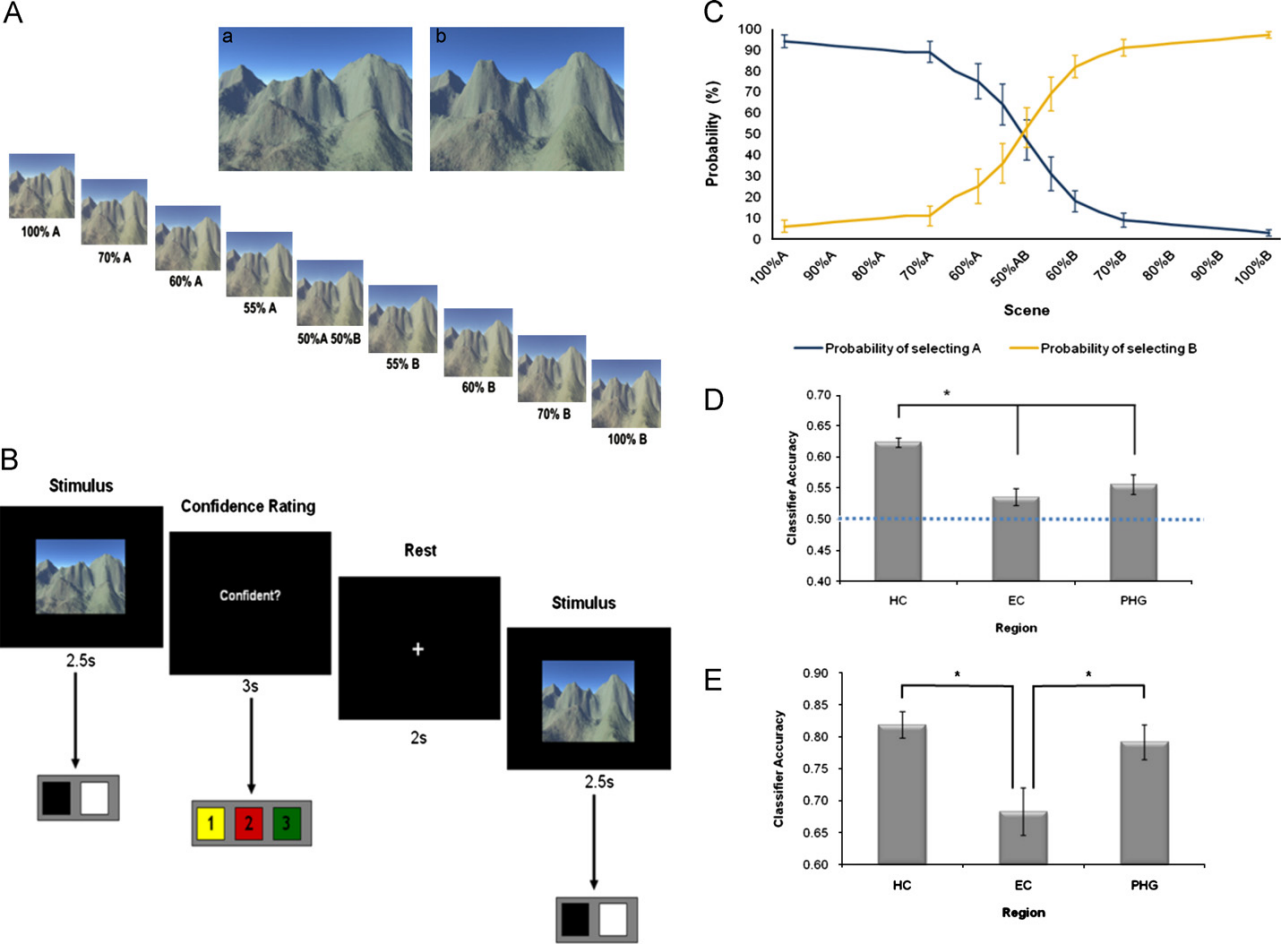
Decoding scene representations in the medial temporal lobe (Bonnici et al., 2012). (A) The two original scenes are displayed at the top, along with full set of morph stimuli below (note the labels A and B were never used in the actual experiment). (B) Timeline of a single trial comprised a stimulus duration of 2.5 s during which the participant registered their decision. Participants then indicated their confidence in that decision during the next 3 s, from a choice of sure, fairly sure, and very sure. There was then a 2 s rest before the start of the next trial. (C) Group data showing the choice behaviour for each of the morph stimuli, which clearly followed a sigmoid profile. (D) Average classification accuracy values for the original (100%) scenes displayed for the hippocampus (HC), entorhinal cortex (EC) and posterior parahippocampal gyrus (PHG). For all three regions classification accuracy was significantly above chance, although the HC classifier performed significantly better than the EC and PHG classifiers (*p<0.05). (E) Average classifier accuracy values for the 50% morphed scenes (i.e., perceptually ambiguous). Classifier performance was significantly above chance in all three regions, with the HC and PHG classifiers both out-performing that of the EC (*p<0.05).

In the previous study, the hippocampus was found to be involved in pattern separation processes for overlapping episodic-like memories, but also with supporting general representations of spatial context shared across different memories. This latter result is consistent with a second important computational role of the hippocampus whereby ambiguous or partial inputs automatically induce the retrieval of the complete neural pattern through pattern completion (Marr, 1971; Treves & Rolls, 1994; O'Reilly & Rudy, 2000). Bonnici et al. (2012), set out to further investigate the role of the hippocampus in pattern separation and pattern completion in a simple decision-making task involving highly overlapping scenes. Their first question was whether the hippocampus maintains distinct representations of scenes that are highly overlapping in terms of their perceptual features, which would provide further support for the role of the hippocampus in pattern separation. In order to address this question, two similar scenes (A and B) were created, which permitted total control over the perceptual features of the two stimuli. As can be seen in Fig. 5A, the two scenes were highly similar.

The second question was whether there was evidence for pattern completion processes in the hippocampus. In order to investigate this, a series of morphs spanning a range of similarities between original scenes A and B were created (Fig. 5A). The morphed stimuli allowed Bonnici et al. (2012) to probe the pattern of activity expressed within the hippocampus to each morph, and whether responses were consistent with pattern completion processes. Prior to scanning, sixteen participants learnt which of two actions was rewarded (e.g., a right button push) in relation to each of the two scenes A and B, receiving monetary feedback for the correct responses. Once they had learned this discrimination, participants were scanned while they performed a new discrimination task involving the original scenes A and B plus all of the morphed scenes. Although the morphed scenes themselves were not linked to reinforcement at any stage, the participants were instructed to select the action most likely to yield monetary reward for each scene, and then to make a confidence rating in that decision. During this phase there was no feedback following the decisions (Fig. 5B).

The first question of interest was whether it was possible to differentiate the two original scenes A and B based on patterns of activity within the hippocampus and surrounding MTL. In order to do this, the hippocampus, entorhinal cortex, and posterior parahippocampal gyrus were delineated as ROIs, and MVPA analysis applied to each. The particular MVPA algorithm used was the same as that described above in Chadwick et al. (2010). Evidence for distinct scene representations was found within all three regions of interest, despite the high level of similarity between the two scenes, demonstrating that information about specific scenes is widely distributed throughout the MTL. However, the classification accuracy in the hippocampus was significantly higher than the other MTL regions (Fig. 5D). These results, therefore, provide evidence that the hippocampus maintains distinct representations of complex scenes through pattern separation, dovetailing with previously observed scene discrimination deficits in rodents and humans with hippocampal damage (Graham et al., 2006; McHugh et al., 2007). It should be noted that an earlier MVPA study by Diana, Yonelinas, and Ranganath (2008) failed to find evidence for scene representations within the hippocampus, but did find it within parahippocampal cortex, which might appear to be at odds with the Bonnici et al. (2012) study. However, there is an important difference between these two studies—Diana et al. (2008) analysed information at the level of stimulus categories, rather than the individual scene representations which was the focus of Bonnici et al. (2012) (for further discussion of this point, see Section 3).

The second question was whether pattern completion was detectable in fMRI responses to the set of morphed scenes. The first test of this was based on the 50% morphs, where the perceptual properties of the stimulus were equidistant from both of the original scenes. Behaviourally, the participants tended to categorise these morphs as scenes A and B equally often (Fig. 5C), but interestingly, these choices were accompanied by a relatively high level of confidence in the decisions, suggesting that they were not merely guesses. This is important, because it allowed Bonnici et al. (2012) to investigate whether there was any information in the hippocampus that permitted differentiation of the decision states A and B when the visual properties of the stimulus were exactly matched (i.e., it was always the same 50% morph). If there were distinct patterns of activity for these decision states, then this would provide evidence for a pattern completion process, whereby a perceptually ambiguous stimulus was “pattern completed” into one of two decision categories, leading to a participant confidently asserting that the stimulus belongs to one category over the other. For this analysis, Bonnici et al. (2012) looked exclusively at the 50% morphs, and attempted to classify them based on the participants' choices. Interestingly, all three MTL regions contained a significant amount of information about the participants' choices even when the stimuli themselves were exactly the same. A comparison of the accuracy values across the three regions found that both the hippocampus and parahippocampal gyrus performed significantly better than the entorhinal cortex (Fig. 5E).

They then further probed the specific computations involved in the representations of the morphed scenes within the hippocampus. One possibility is that the hippocampal representations expressed during the 50% morph trials reflect reinstatements of the original learnt scenes A and B, a process consistent with the operation of a bistable attractor network (i.e., a neural network where new inputs will be categorised as one of two possible representations based on their similarity to each). Neural network models of hippocampal function have often proposed the view that memories are stored as discrete local attractors (Hopfield, 1982; Treves & Rolls, 1994), with partial or ambiguous inputs inducing the network to abruptly move into one of those discrete states through “global” pattern completion. In order to test this idea, an MVPA classifier was trained to differentiate the original scenes A and B, and tested it on the 50% morphs. If the ambiguous morph stimuli are inducing hippocampal ensembles to shift into one of two discrete attractor state representing scene A or B, then this should produce significant decoding results within the hippocampus. However, this analysis did not produce significant results in any of the three MTL regions, which argues against this interpretation.

The failure to find evidence of discrete, bistable attractor networks within the hippocampus may not be entirely surprising given previous work in rodents where the operation of attractors may depend on the exact experimental parameters imposed (Leutgeb, Leutgeb, Moser, & Moser, 2005; Wills et al., 2005). Bonnici et al. (2012) therefore investigated an alternative scenario in which hippocampal ensembles respond to ambiguous inputs (50% morphs) by transitioning to intermediate attractor configurations through a more limited form a pattern completion. An MVPA classifier was trained to differentiate the 50% morph trials based on the decisions made on each trial, and then tested on all of the other morph trials (i.e., the 55%, 60%, and 70% morphs—see Fig. 5). The results of this analysis showed that the hippocampus could successfully generalise between the subjective decision made on viewing the 50% morphs, and those made on the other morph scenes (55–70% morphs), while this was not the case in the other MTL regions.

This result suggests that in this simple binary decision task, hippocampal ensembles may have configured into one of four distinct attractor states, which appeared to correspond to something like ‘scene A’, ‘scene B’, ‘A-like’, and ‘B-like’. The first two states were induced by the original scenes A and B which were over-learned prior to scanning, and therefore were highly stable representations. The second two states were induced by the morphs, and would seem to suggest that the hippocampus “knew” that the morphs were not exact replications of scenes A and B, and therefore did not simply assign them to these two pre-existing attractor states. Instead, it appears that two new attractor states were created based on whether the morphs were most “like” scene A or scene B. These attractor states were subsequently applied to all the morphs, including the completely perceptually ambiguous 50% morphs, leading to the result that Bonnici et al. (2012) could decode these morphs based on the decision of the participants. Thus, they ended up with two “absolute” attractor states for the original scenes A and B, and two “intermediate” attractor states where the ambiguous scenes were known to be distinct from the original two scenes, but nevertheless could be accurately categorised based on similarity to one or the other.

This insight into the computational operations of the hippocampus suggests that attractor dynamics may be more complex than simply assuming that the number of attractors will be equal to the number of perceptual decisions a subject has to make (in this case, is the scene more like A or B?). Instead, there may be multiple attractor states based on the interaction of the perceptual properties of stimuli and the motivations and goals that are relevant to the task at hand. Altogether this raises the interesting possibility that hippocampal attractor states could play a more active role in decision-making than previously thought, and may act as intermediate representations between purely perceptual states and the goal or decision states required for high-level decision-making.

### 2.5 Interim summary and conclusions

To date, MVPA analyses have revealed that it is possible to decode complex, realistic information such as allocentric spatial locations within a virtual environment (Hassabis et al., 2009; Rodriguez, 2011) and episodic-like memories (Chadwick et al., 2010) from patterns of activity across voxels in the human hippocampus. Using MVPA to probe the nature of hippocampal representations more closely, evidence emerged that the hippocampus maintains distinct representations of highly overlapping episodic-like memories, providing a link between theories of pattern separation and complex episodic memories (Chadwick et al., 2011). Similarly, Bonnici et al. (2012) found that the hippocampus maintains representations of highly similar scenes, and furthermore, that even when perceptual inputs were held entirely constant (in the 50% morph trials), the patterns of hippocampal activity showed a robust relationship with participants' decisions, suggesting the hippocampus may play a more active role in decision-making than previously thought.

This set of studies gives a flavour of the kinds of questions that can be addressed with the use of MVPA analyses when focused on the hippocampus and MTL. As alluded to previously, it is important to note that standard mass-univariate analyses were conducted on all of the data sets described above and none provided any significant results, demonstrating that MVPA may be an important alternative approach for interrogating the informational content of brain regions like the hippocampus in some circumstances. As such, while the number of MVPA studies investigating information in the hippocampus is currently small, we hope that the results so far will encourage greater use of this approach in the future, as it may prove valuable for gaining new leverage on some long-standing debates within the hippocampus literature.

## 3 Frequently-asked-questions about MVPA

Having outlined some examples of MVPA in action, in this section we consider a variety of questions that have cropped up in the course of the studies described in Section 2. The hope is that we may offer some guidance to others in the use of MVPA to study the hippocampus and memory (and indeed in other domains as well, as the issues discussed here have general relevance to MVPA and fMRI). Several excellent reviews of MVPA analysis in theory and practice already exist (Haynes & Rees, 2006; Norman et al., 2006; Mur, Bandettini, & Kriegeskorte, 2009; Pereira, Mitchell, & Botvinick, 2009; Weil & Rees, 2010; Rissman & Wagner, 2012), so we do not offer another here. Rather, the purpose of this section is to give a practical overview of the types of issues that may need to be considered when implementing MVPA.

### 3.1 Which MVPA method should I use?

There are many different MVPA algorithms to choose from, prompting the obvious question, which one is best? One of the most widely used algorithms is the linear support vector machine (SVM) as it is a powerful and sensitive multivariate tool and easily accessible. This is the type of algorithm used in the studies described in Section 2. The particular implementation was libsvm, which is a freely available, easy to use library of SVM tools compatible with multiple platforms (Chang & Lin, 2011). Other algorithms include: linear discriminant analysis, nearest neighbour, naïve bayes, multinomial logistic regression, and classification trees, to name just a few. Several studies have directly compared the performance of different MVPA algorithms (Cox & Savoy, 2003; Mitchell, Hutchinson, Niculescu, Pereira, & Wang, 2004; Ku, Gretton, Macke, & Logothetis, 2008; Misaki, Kim, Bandettini, & Kriegeskorte, 2010), the most comprehensive of which was a comparison of classification algorithms and pre-processing methods by Misaki et al. (2010). While slight advantages of some algorithms over others were found, there was not a great deal of consistency between the findings, suggesting that algorithm performance may depend on the particular dataset used. Overall, there is no clear evidence suggesting a strong benefit for any type of MVPA algorithm over others at this time. One notable limitation of all these techniques is the need to train the algorithm, which necessitates the repetition of the stimulus classes multiple times during the experiment. In many cases, this is not a problem, as the representations themselves are expected to remain relatively stable over multiple repetitions. However, in domains such as learning/encoding, where the dynamic change in representations over time is important, it would be desirable to investigate the representational properties of individual stimuli without having to present them multiple times. This is currently not easily accomplished using MVPA algorithms such as SVM.

Another commonly used MVPA approach is representational similarity analysis (Kriegeskorte et al., 2008a, 2008b). This is based on the simplest kind of multivariate inference one can make—taking the pattern of voxel activation elicited by two different stimuli, and measuring the multivariate distance between these two patterns using a simple measure such as a correlation. Despite the simplicity of this method, when appropriately applied, this type of analysis can reveal information about the structure of representations with a good deal of flexibility. Kriegeskorte et al. (2008b) demonstrated this in a comparative study of object representations within human and monkey inferior temporal cortex. In addition to comparing different species, different techniques were used in both species, with fMRI data collected from the human participants, and electrophysiological data from the monkeys. A large number of stimuli were presented to both species, and a correlation was calculated for each pair of stimuli. This was derived from the pattern of voxels in inferior temporal cortex in humans, and from the spiking activity across multiple electrodes in the monkeys. This step effectively abstracted the information from data that was species and technique-specific, to data that was coded in terms of the similarity relationship between each pair of stimuli. This abstraction allowed them to compare the representational structure of the many stimuli (now represented as a correlation matrix, or “similarity matrix”) across the species. They found a striking correspondence in the similarity matrices between the species, indicating that both humans and monkeys may code visual stimuli in a similar way within inferior temporal cortex, thus demonstrating the potential power of this approach.

Because representational similarity analysis rests on a conceptually simpler approach than the more complex MVPA SVM algorithms, it can lend itself to easier interpretation of the results, as well as potentially providing more flexibility in exploring the relationships between different representations. Another advantage is that pair-wise correlations do not require multiple stimulus repetitions. It is therefore possible to investigate the representational properties of stimuli that are presented only once, which could potentially be an advantage for certain experimental questions. On the other hand, this approach is likely to be less sensitive than more complex algorithms when investigating subtle differences in multivariate data.

Another promising recent development is a Bayesian model-based approach to decoding called Multivariate Bayes (MVB) that is implemented in SPM. MVB maps multivariate voxel responses to a psychological target variable (e.g., individual memories), using a hierarchical approach known as Parametric Empirical Bayes (Friston et al., 2008; Morcom & Friston, 2012). MVB uses the same design matrix of experimental variables used in a conventional SPM analysis. When a decoding contrast is specified, a Target variable *X* is derived from this contrast, after removing confounds. The multivariate voxel activity provides the predictor variable *Y*, which the MVB model will try to fit to *X*, ultimately producing a log model evidence, or Bayes factor for that model. The log evidence can be considered as a measure of the mutual information between the multivariate data and the psychological variable. There are several potential advantages to the use of this kind of model-based approach over other methods such as the SVM. First, by explicitly modelling the mutual information in this way, MVB is potentially more sensitive to the underlying neural representations. Second, this method does not require the train-test, cross-validation approach in order to assess the underlying information, as this is provided by the log evidence for the model. Third, because the multivariate data from a region is explicitly formulated as part of the model in an MVB design, it becomes possible to directly compare information across different regions, as this now reduces to a model comparison (although note that it may be necessary to adjust the log-evidence to account for differences in ROI size). This point is particularly important for MVPA analysis, as comparing the results of other MVPA methods across different regions can be problematic. The reason for this is that there may be regional differences in signal-to-noise ratio that affect the decoding results. Thus, differences in classification accuracy between two regions may not reflect genuine distinctions in the underlying neural information in these brain areas. MVB, on the other hand, incorporates such regional effects within the model itself, thereby providing a valid means of directly comparing information across different regions.

An important caveat is that MVB analysis uses an explicit prior that there is a meaningful mapping between the experimental variable and the data. If the aim is to explicitly compare the information contained within different regions then this is not a problem, as the evidence associated with the two models (regions) can be directly compared. However, if the aim of the analysis is simply to determine whether or not there is a significant amount of information in the underlying multivariate signal of a single region, then the log-evidence furnished by MVB should not be used to assess this, as it may be biased in favour of a positive result. In cases such as this a classical statistic can be derived through permutation testing, or alternative MVPA methods can be used instead. Overall, however, the MVB approach is a potentially attractive method for multivariate decoding of fMRI data, particularly when it is desirable to directly compare information between regions.

Gallant and colleagues (Kay, Naselaris, Prenger, & Gallant, 2008; Naselaris, Prenger, Kay, Oliver, & Gallant, 2009; Naselaris, Kay, Nishimoto & Gallant, 2011) have developed another powerful model-based approach to decoding over the last few years. This group uses a voxel encoding approach, whereby theories about the activity of underlying neuronal populations are used to model the response of each voxel to stimuli. For example, Naselaris et al. (2009) modelled the response of voxels in early visual cortex based on evidence that early visual cortex represents visual stimulation in three low-level domains—orientation, spatial frequency, and spatial location. The tuning of each voxel to these domains was modelled using a Gabor wavelet method. Using the fully trained model, they were able to reconstruct novel scenes solely on the basis of the pattern of activation across visual cortex. In addition to being theoretically important for testing models of neural representation, the ability to investigate the neural response to novel stimuli provides a significant practical advantage over MVPA approaches such as SVM which, as alluded to above, must be trained on multiple repetitions of each stimulus class. However, while the voxel encoding approach has proven successful in early visual cortex where there are well-defined models of neuronal population dynamics, it will be a significant challenge to apply the same approach to higher-level regions such as the hippocampus. The reason for this is that it is not clear precisely what properties could be usefully modelled in a hippocampal voxel. While a V1 voxel has a relatively constrained set of variables that it can respond to, and can be modelled in terms of just three dimensions (orientation, spatial location, spatial frequency), the number and type of possible variables that a hippocampal voxel could respond to is vastly more complex. Thus, effective use of this method in the hippocampus will require the definition of a much more constrained set of invariant features that can comprehensively define the response of a single hippocampal voxel.

Ultimately there is no right or wrong answer to the question of what MVPA method to adopt, but a pragmatic approach would be to consider how distinct the representations are likely to be—if you expect them to be relatively distinct, then a simpler multivariate approach such as representational similarity analysis may be suitable, due to its ease and flexibility of interpretation. If, however, the differences between the representations are likely to be quite subtle, then a more complex algorithm such as an SVM will probably be more appropriate.

### 3.2 How should I pre-process my data?

As well as a choice of MVPA algorithms, there are also a variety of approaches to data pre-processing. Early MVPA hippocampal studies (e.g., Hassabis et al., 2009; Chadwick et al., 2010) generally extracted the raw BOLD signal (after correcting for linear or nonlinear signal drift) at around 6 s following the onset of the stimuli, and used this raw signal as the MVPA input. This approach has had a good deal of success, but does not attempt to model the haemodynamic response function (HRF), and may therefore be ignoring important information. An alternative approach is to explicitly model the HRF for each trial in a general linear model (GLM), and use the resulting parameter (beta) estimates as the MVPA input. This approach has also proved successful in a number of studies including that of Bonnici et al. (2012), described earlier. More recently, a comprehensive comparison of various MVPA analysis steps by Misaki et al. (2010) suggested that using *t*-values based on GLM beta estimates (by dividing the beta by its standard error estimate) produced optimal MVPA results, and this was the approach adopted in the Chadwick et al. (2011) study. For a comparison of different pre-processing approaches to event-related MVPA, see Mumford, Turner, Ashby, and Poldrack (2012), who also concluded that using a form of *t*-value produced optimal results. In summary, the pre-processing method of choice at present appears to be the use of the GLM to produce *t*-values as the input to MVPA analyses. However, it is important to note that this does not invalidate the use of other approaches such as raw BOLD or betas, rather the evidence suggests that these approaches may be sub-optimal, reducing the power of the analysis, making it more difficult to observe significant results.

Each of the procedures described above should first include a motion correction processing step, whereby the individual functional images are realigned to one another, as in mass-univariate analysis. Additionally, slice time correction can be applied as in a standard mass-univariate analysis. The choice of whether or not spatial normalization is applied to the data depends on the specific MVPA approach used. If the analysis is constrained to anatomically-defined ROIs, then it is preferable to analyse the data in each subject's native-space, in order to miminise the spatial distortion of the data. If, however, a searchlight approach is used, then the data should be spatially normalized as in a standard mass-univariate analysis, so that the location of information can be assessed at the group level. The final pre-processing step usually applied in a mass-univariate analysis is spatial smoothing. The majority of MVPA studies to date have either used minimal smoothing, or omitted smoothing altogether, in order to preserve the fine-grained spatial patterns of information. Some have suggested that spatial smoothing does not harm MVPA performance (Op de Beeck, 2010), but note that our experiences suggest that this may depend on the particular dataset in question (see Section 3.6 for more details on this point). Thus, we would suggest a conservative approach with smoothing, using minimal smoothing or none at all.

### 3.3 Should I use a whole-brain analysis, ROIs, or a searchlight approach?

One of the critical choices to be made in an MVPA analysis concerns the initial selection of data to be analysed. Broadly speaking there are three main choices: (a) MVPA within a given ROI (b) MVPA based on activity across the whole brain, and (c) searchlight-based MVPA (Kriegeskorte, Goebel, & Bandettini, 2006). Each of these approaches is entirely legitimate, and the optimal choice will depend on the particular dataset and the question of interest.

The most common approach is to employ regions-of-interest, where the multivariate information is assessed within a specific brain region, which can be defined either anatomically or functionally (e.g., using a functional localiser). Once the region is designated, the activity is extracted from all voxels within that ROI, and an MVPA analysis is applied in order to interrogate the patterns of information present within this set of voxels. This approach can be useful for hypothesis-driven research (e.g., in the hippocampus), as it allows one to draw specific conclusions about the informational content of a particular brain area. However, it is important to note that ROI analyses, when based on functionally defined regions-of-interest, require researchers to exercise care to ensure that they do not fall foul of “double-dipping”. This is where the same data are used for selection and further in-depth analyses in a biased fashion. For details on how to avoid this error, see Kriegeskorte, Simmons, Bellgowan, and Baker (2009), and Pereira et al. (2009). When used appropriately, ROI-based MVPA analyses can provide evidence about localised information processing in a way that is not possible with standard mass-univariate analyses.

The second approach involves investigating multivariate information that may be widely distributed across the whole brain. In these analyses the voxel activity is extracted from the whole brain (or a large part of it, such as the grey matter), and MVPA is applied to this entire set of voxels. This type of analysis is therefore not anatomically specific, but instead examines the multivariate information present across patterns of voxels that may be widely distributed across the brain. There are two potential problems surrounding the use and interpretation of whole-brain decoding analysis. First, because so many voxels are included in the analysis, this approach will only be successful when the brain states are quite distinct, and will likely not work as effectively for more subtle information present in localised regions of the brain. Second, determining the location of information from a whole-brain MVPA is not straightforward, due to the inherently multivariate nature of the analysis (Friston, Frith, Frackowiak, & Turner, 1995). Therefore, this method is most appropriate when mapping information between cognitive states and neural activity as a whole, and where the precise location of that information is not the critical question of interest. An excellent example of the type of questions that are best addressed with whole-brain decoding was a study by Polyn, Natu, Dohen and Norman (2005). Using this method they found that information about stimulus category was present in the brain-wide pattern of voxel activation during free recall of individual stimuli. Furthermore, this category-level information was actually present prior to the retrieval of individual items, indicating that some form of context-dependent retrieval may have helped the participants to recall the specific items. Importantly, the purpose of this study was to map brain-wide activity patterns to cognitive states in order to inform theories of cognition rather than to localise information within the brain. It is precisely this kind of question that can be most usefully informed by the application of whole-brain MVPA.

The third common method is known as “searchlight” analysis (Kriegeskorte et al., 2006), which is a means of assessing local multivariate information across large areas of the brain, or even the whole brain. In a searchlight analysis, a “searchlight” region of interest is created for every single voxel across the brain. Each searchlight consists of a sphere of voxels (typically around 100 voxels, depending on the voxel resolution) surrounding the central voxel. A separate MVPA analysis is applied to each of these searchlights, creating an accuracy value for every single voxel in the brain, which can be displayed as an “accuracy” or “information map”. A statistical threshold can then be applied to this map at either the single subject or group level in much the same way as a mass-univariate analysis. This method therefore allows one to search over the whole brain for information carried in the local multivariate response patterns. This approach is in some ways an intermediate between the other two forms of analysis. Searchlight analysis (Kriegeskorte et al., 2006) effectively applies local ROI-based MVPA analysis across the entire search space, which could include the whole brain (or the cortical surface as in recent surface-based searchlight approaches—see Oosterhof, Wiesterl, Downing, & Diedrichsen, 2011). This method is probably the most appropriate for exploratory analysis of representations across the whole brain (or a portion of it), and allows for the localisation of information in a way that whole-brain MVPA does not. The major draw-back of the searchlight approach is that the many thousands of MVPA analyses lead to the problem of multiple comparisons that is also inherent in mass-univariate analysis, and the same procedures should be used to correct for this.

In summary, ROI-based analyses are often appropriate for hypothesis-driven MVPA analyses or for additional MVPA analyses within the context of mass-univariate studies, but for more exploratory analyses, searchlight MVPA is preferable for its ability to accurately localise multivariate information across the whole brain. Whole-brain MVPA is most useful when the question of interest regards a mapping between cognitive states and widely distributed neural activity.

### 3.4 Should I use feature selection?

Within a given dataset, it is likely that some voxels will not carry any useful information about the representations of interest, only adding noise to the MVPA analysis. A frequently used method within MVPA research is “feature selection”, whose purpose is to reduce a set of voxels to those that are most likely to carry information (or inversely, to remove those voxels most likely to carry noise). There are many approaches to feature selection (Guyon & Ellisseeff, 2003), that we cannot cover here, but the majority involve two basic steps. In step 1, the informational content of each voxel is assessed, and the set of voxels is ranked accordingly. Step 2 then involves the application of a threshold criterion to these ranked data in order to select the set of voxels most likely to contain information. Finally, this reduced set is used as the input to the MVPA analysis. The approach used in some of the studies described in Section 2 involved a searchlight MVPA to assess the local information at each voxel within an ROI. A related approach to reducing noise in a dataset is “feature reduction”, also known as “dimensionality reduction”. In this approach, the aim is not to remove individual voxels from the analysis, but instead to summarise the dataset in a smaller number of features using approaches such as principal component analysis or independent component analysis. In this case, the input to the MVPA algorithm is no longer the set of individual voxels, but instead the set of principal/independent components, which may help to reduce noise. However, it is important to note that neither approach to feature selection/reduction is perfect, and there is no guarantee that important information will not be lost during a feature selection or reduction step. Thus, it will not always be advantageous to use feature selection. For a thorough discussion of this issue and related methodological points, see Pereira et al. (2009).

### 3.5 Should I use MVPA or an fMRI adaptation paradigm?

Another method of fMRI analysis that has been used to probe neuronal processes at the level of representations is fMRI adaptation (fMRIa). This examines the effect of repeating stimuli over time with the hypothesis that stimuli that activate overlapping neuronal representations will elicit a reduced response, for which there is substantial evidence (Grill-Spector, Kourtzi, & Kanwisher, 2001; Kourtzi & Kanwisher, 2001; Grill-Spector, Henson, & Martin, 2006). Recent findings suggest that, while MVPA and fMRIa may both provide a means of tapping into information at the level of individual representations, they may be sensitive to different types of neural signal. For instance, Drucker and Aguirre (2009) directly compared MVPA and fMRIa in an object shape task, and found a double dissociation within the lateral occipital complex (LOC), with ventral LOC showing adaptation effects, and lateral LOC showing decoding effects. The interpretation of these results was that decoding analyses are more sensitive to information coded by narrowly tuned neurons clustered by their response properties, whereas adaptation is more sensitive to information coded by broadly tuned neurons with no clustering principle. Similarly, Epstein and Morgan (2012) found interesting distinctions between MVPA and fMRIa analyses of scene and landmark representations. Together, this evidence suggests that MVPA and fMRIa are not simply interchangeable approaches, and may provide complementary insights into information processing.

### 3.6 Is it better to use high-resolution fMRI for MVPA?

A current debate in the MVPA literature concerns the level of information being detected by this technique (Kamitani & Sawahata, 2010; Op de Beeck, 2010; Swisher et al., 2010; Freeman, Brouwer, Heeger, & Merriam, 2011), and one of the practical questions arising from this debate is whether high-resolution fMRI is necessary for MVPA analysis. There are many studies that have reported robust MVPA results using standard resolution fMRI (e.g., 3 mm isotropic voxels), including some of the earlier visual studies (e.g., Haynes & Rees, 2005; Kamitani & Tong, 2005), which demonstrates that high-resolution is not a prerequisite for all decoding analyses. However, the question of whether high-resolution scanning can increase the power of MVPA analyses is still an open question, and has not been fully explored with regard to hippocampal representations. Another complicating factor is that higher-resolution scanning necessitates longer acquisition times, and a potential tradeoff with signal-to-noise-ratio (Carr et al., 2010). Nevertheless, all of the studies described in Section 2 used a high-resolution (1.5 mm isotropic voxels) fMRI acquisition sequence focused on the MTL, with the assumption that by maximising the spatial resolution within a region of interest, information would be maximised. This is only the case, of course, if there are significant sources of information available at a finer spatial scale then are available in coarser-resolution data. An indirect source of evidence comes from Rodriguez (2011), who found evidence for some spatial information within the hippocampus using a standard voxel resolution. This suggests that high-resolution might not be a requirement for MVPA analyses of the hippocampus. However, Chadwick et al. (2011) compared different levels of smoothing (thus effectively altering the resolution of the underlying information) in an MVPA analysis of episodic memories focused on the hippocampus, and found that greater smoothing (i.e., coarser resolution) adversely affected MVPA performance (while 3 mm smoothing was still effective, 6 mm smoothing and above significantly decreased MVPA performance). Altogether, there is no clear answer to this question, as it appears that high-resolution data are important in some circumstances and not in others. The most parsimonious approach, therefore, would seem to be to consider carefully whether the experimental hypothesis is anatomically specific (e.g., focused on the MTL), or whether it is more exploratory in nature and/or is expected to involve more distributed neural regions. If it is the former, then it would seem sensible to acquire high-resolution data in order to maximise the information gathered from that region. If the latter, then it would be more appropriate to acquire lower resolution data in order to cover the whole brain.

### 3.7 Why are my classification accuracies not near 100%?

There is a large amount of variability in the level of classification accuracy reported in the MVPA literature, with some studies describing impressively high classification rates of 80% or more. Thus, one common question concerns what level of accuracy should be considered meaningful. It is important to note that the level of accuracy that it is possible to achieve in any given study depends heavily on the complexity of the information being decoded. When two representations are highly separable, such as faces and places, then it should be possible to classify them with a high degree of accuracy. If, however, the representations are more complex (such as autobiographical memories), or more similar to one another (such as two very similar scenes) then the patterns of activity relating to each representation may be more difficult to separate, and the MVPA classification accuracy will be lower. In some circumstances, therefore, it is not reasonable to expect high levels of classification accuracy. Ultimately, however, what is relevant is not so much the absolute level of accuracy achieved, but whether the results are robust enough to be statistically significant, and replicable.

### 3.8 Why does MVPA have to be multivariate?

The question here is whether in theory, and given sufficient power, similar inferences could be drawn using the signal from a single voxel? Theoretically, the answer is yes. Given a voxel which is consistently active for one memory compared to another, we could easily use this to differentiate two individual memories. We could even “train” an algorithm on a portion of data and use it to “test” an independent test set, in a similar fashion to standard MVPA classification (although in fact this would be unnecessary if all we wish to do is to infer a significant mapping between activity and the experimental variable, as we could use classical statistics). Practically speaking, however, the use of a multivariate approach can greatly enhance the sensitivity of the analysis to underlying information, by combining information across many voxels. This property is extremely advantageous when dealing with real fMRI datasets that are likely to be noisy. Furthermore, if one were searching for individually informative voxels within a specific ROI (such as the hippocampus), one would have to test many hundreds of voxels independently, and then correct the subsequent *p* values for multiple comparisons. The MVPA equivalent would only involve a single statistical test, thereby avoiding the multiple comparisons problem. Overall, therefore, there are clear practical reasons why MVPA methods have come to the fore when investigating subtle cognitive representations.

### 3.9 Can I examine different levels of representation?

Beyond all of the methodological choices and challenges, the most critical element of any MVPA study is proper consideration of the level of representation being decoded, and from that, making appropriate inferences about the underlying neural processes. There are three levels of representation that have been investigated using MVPA, the broadest of which we will term “cognitive state”. MVPA analyses of cognitive states are those that investigate processes rather than specific stimuli/representations. An example of this is evident in a study by Rissman, Greely, and Wagner (2010) who demonstrated that it is possible to decode subjective mnemonic states (e.g., a feeling that a face is new or old) from whole-brain patterns of voxel activity. This MVPA analysis is not specific to any particular type of representation, but instead is related to the cognitive states relating to recognition memory. It is worth noting that this level of representation is not uniquely accessible to MVPA analyses, as the mass-univariate approach was originally developed in order to differentiate cognitive states, although MVPA may offer a more sensitive measure of information in some circumstances.

Stimulus categories, such as faces or places (or nouns and verbs) constitute another level of representation, and MVPA has been useful for elucidating category-level information. Again, this level of information is available to mass-univariate analyses, as exemplified by studies investigating the “fusiform face area” and “parahippocampal place area” (Kanwisher, McDermott, & Chun, 1997; Epstein & Kanwisher, 1998). However, as originally demonstrated by Haxby et al. (2001), there may be residual information about non-preferred categories within each of these regions when analysed with MVPA, which demonstrates that both approaches are necessary for a full understanding of the neural representation of categories.

The most detailed level of representation is the item-level, where MVPA is used to investigate the representation of individual stimuli (e.g., memories). This is the only level of representation where MVPA is essential, as it is not usually possible to find a regional difference in overall activation between two individual scenes or faces, using a mass-univariate analysis. All of the MVPA analyses of the hippocampus described in Section 2 involved investigating item-level representations, whether it be specific spatial locations (Hassabis et al., 2009), individual episodic memories (Chadwick et al., 2010, 2011), or individual scenes (Bonnici et al., 2012). A comparison of two MVPA studies of the MTL illustrates the different (but complementary) interpretations that can be made from investigating different levels of representation. Diana et al. (2008) used MVPA to investigate the representation of various stimulus categories (including scenes) in both the posterior parahippocampal gyrus and the hippocampus. They found evidence of category-level information within the former but not the latter. Bonnici et al. (2012) investigated the representation of individual scenes within the MTL, and found evidence for scene representations within both the parahippocampal cortex and the hippocampus. This suggests the hippocampus may contain more distinct representations of individual scenes which are not organised in a category-specific fashion, thereby allowing successful item decoding but not category decoding.

### 3.10 What am I decoding?

Finally we want to emphasise that ultimately, as with any neuroimaging approach, the interpretation of the results is always constrained by the experimental design. The results of the two studies investigating the representation of episodic-like memories (Chadwick et al., 2010, 2011) illustrate this point. In the first study, it was possible to predict which of three episodic-like memories was being recalled from patterns of activity within the hippocampus (Chadwick et al., 2010). From this it was concluded that individual episodic-like representations could be decoded from hippocampal activity. While this is a legitimate conclusion, it does not tell us precisely what information is allowing that decoding, as the three episodes differed along a variety of dimensions, such as the spatial locations and the people featured in each episode. In order to draw more precise conclusions about the representational make-up of episodic-like memories in the hippocampus, a more fully controlled paradigm was required (Chadwick et al., 2011). The level of additional control allowed Chadwick et al. (2011) to conclude that there are at least two types of representation present in the hippocampus during episodic recall: unique ‘bound’ representations, and general spatial representations. Consideration of the experimental design and what it can allow you to infer about the information you want to decode should be considered at the outset of any MVPA study.

## 4 Future directions

We conclude by highlighting some potential future applications of MVPA that may be particularly relevant to hippocampal research. The first is the use of MVPA to revisit some of the major debates in the hippocampus literature, in order to gain new insights. For example, the ability to examine individual memory representations may offer new leverage on the issue of systems-level consolidation and the timescale of hippocampal involvement in representing episodic/autobiographical memories. The second development is the capacity to investigate activity within the subfields of the human hippocampus using high-resolution MRI (Carr et al., 2010) which, if combined with MVPA, could greatly enhance our ability to interrogate the representations and computations within the hippocampus. Computational theories of the hippocampus make clear predictions about the representations we might expect within the different subfields of the hippocampus. For example, regions CA3 and DG in particular may contain distinct representations of very similar stimuli in line with a role in pattern separation (Bakker, Kirwan, Miller, & Stark, 2008). We believe that the use of MVPA in the subfields of the hippocampus will prove invaluable in our quest to understand the computational underpinnings of episodic memory. Finally, it will be important to consider how fMRI and MVPA can be applied in neuropsychological settings (Mayes, 1988), for example, by testing whether viable memory representations are detectable even in damaged hippocampal tissue.

It is not always necessary or appropriate to employ MVPA to study memory and the hippocampus; it is not better by default, and to deploy fMRI MVPA paradigms properly can be challenging. However, the judicious use of MVPA, where key questions cannot be addressed effectively in other ways, in our view makes MVPA an invaluable addition to the hippocampal armamentarium.

## Funding

The authors are supported by the Wellcome Trust.
